# An evaluation of live porcine simulation training for robotic surgery

**DOI:** 10.1007/s11701-020-01113-3

**Published:** 2020-07-11

**Authors:** Nicholas Raison, Johan Poulsen, Takashige Abe, Abdullatif Aydin, Kamran Ahmed, Prokar Dasgupta

**Affiliations:** 1 MRC Centre for Transplantation, King’s College London, London, SE1 9RT UK; 2 Department of Urology, Aalborg University Hospital, Aalborg, Denmark

**Keywords:** Simulation, Robotic surgery, Live porcine simulation, Surgical training, Survey

## Abstract

To assess the role of live porcine simulation in robotic surgical skills training. A qualitative and quantitative survey was conducted of participants of a live porcine robotic simulation course undertaken in a regional training centre. Data on participants’ experience, robotic surgical ability, the educational impact and outcomes from the course were collected. Thirty-nine participants from four different countries completed the survey. Clinical experience varied; however, prior robotic surgical experience (median 0 cases, range 0–100) and technical ability were low. The perceived usefulness, effectiveness and realism of the training course were all highly scored. Participants rated the most useful course components as port placement and docking, basic robotic skills training and repair of a bladder injury. Training resulted in significant increases in technical ability (*p* < 0.0001). Following the course, 49% of participants continued to either train or perform robotic surgery. This survey demonstrates that live porcine simulation for robotic surgery is a highly valued, acceptable and feasible form of training. The majority of participants were relatively inexperienced but nonetheless significant improvements in technical ability were reported. The results of this survey support the use of live porcine training for robotic surgery.

**Electronic supplementary material:**

The online version of this article (10.1007/s11701-020-01113-3) contains supplementary material, which is available to authorized users.

## Introduction

Animals have long been used by surgeons to practice and develop their skills. Yet with the development of virtual reality (VR) and synthetic models, simulation using live animals has become increasingly uncommon. Animal models offer some of the most realistic training models available to surgeons and their teams. These advantages must be weight against a number of moral, ethical and regulatory limitations. The major advantage of animal models is the anatomical similarities and, more importantly, the verisimilitude of the tissues. Simulation training must engage with the complexity and unruliness of real life [1]. This is uniquely offered by live animal models. A wide range of skills ranging from port placement for optimal exposure, cutting, dissection, coagulation and suturing may be practised. Furthermore, using animals for training accurately simulates bleeding enabling the simulation of intraoperative complications [2]. Especially for complications, the reduced clinical experience as a result of the regulatory change in working time has meant that many residents will not be exposed to rare but major complications during training.

Wet laboratory simulation requires dedicated facilities and personnel to ensure that training can be conducted in accordance with ethical and regulatory requirements. The use of animals for training, as for all animal experimentation, must be carefully considered. The principle arguments against the use of live animals for surgical training encompass the ethical and moral objectives to harming animals. If suitable alternatives exist, it is difficult to justify the use of live animals for simulation purposes. The 2002 statement from the American College of Surgeons outlines a similar position that “wherever feasible, alternatives to the use of live animals should be developed and employed”. The college does go on to say that “now and in the foreseeable future it is not possible to completely replace the use of animals ….[in] education and teaching”. Hence, until simulators are able to realistically model the unruly human body accurately, a role for live animal training will continue. For laparoscopic and robotic training, porcine models are most commonly used given the anatomical similarity to humans. Yet to date, only limited assessment of the role of live animal simulation has been undertaken. This study aimed to evaluate the role of live animal simulation for robotic surgical training through a qualitative and quantitative survey of participants at a live porcine robotic training course.

## Methods

### The live porcine robotic simulation course

The porcine robotic training programme costs of a two-day hands-on training course held at the Minimal Invasiv Udviklings Center, Aalborg University Hospital, Aalborg, Denmark. The course was developed with a focus on hands-on training and was primarily aimed at surgeons with at least an intermediate level of skill in robotic surgery. The programme includes a mixture of short lectures on technical performance interspersed with sessions of robotic training which comprises the bulk of the course. Participants are divided into pairs alternating between working on the console and assisting. Proctoring is provided by an expert robotic surgeon and an experienced bedside assistant is present.

The course is held in the biomedical research laboratory; a purpose-built unit with the necessary facilities and equipment to provide safe and ethical care for the animals. Alongside surgical training, the laboratory also conducts regular scientific studies involving animals such as domestic pigs, mini-pigs, rabbits, guinea pigs, rats and mice. As a result, laboratory staff have extensive experience in caring for animals. Official approval is provided by the Danish Animal Experiments Inspectorate. Throughout each course an experienced veterinarian manages the animals, providing anaesthesia, physiological support and eventual euthanasia.

### Development of the participant survey

An online evaluation of participants who had completed the course over the preceding 10 years was conducted. A questionnaire was developed in cooperation with surgical training experts (Supplementary Fig. 1). It consisted of 18 questions focussed on the participants prior robotic surgical experience, self-assessed level of robotic skill before and after completing the training programme, and a comparative assessment of the training course. Respondents were asked to self-assess their surgical ability and confidence out of 100. 5-point Likert scales were used to rate the educational benefits of the individual course components. Participants were contacted directly by email and invited to complete the survey using an internet link. Two reminder emails were sent to all participants who did not reply.

### Statistical analysis

Data analysis and graphs were plotted using GraphPad Prism version 8.0 for macOS, GraphPad Software, La Jolla California US Results. Self-assessment scores were compared using Mann Whitney *U* tests. Correlations between final assessment scores were analysed using multiple linear regression analysis.

## Results

### Baseline participant characteristics

One hundred and seventy-four participants attended the course between 2009 and 2014. Contact details were available for 140 people all of whom were invited to take part in the survey. Thirty-nine (28%) of contacted participants responded and completed the full survey. Participants experience ranged from attending surgeons to year 1 Residents. 49% of participants were early years residents (years 1–3), 36% were senior residents (year 4 +), and 15% were attending surgeons. 72% (*n* = 28) of respondents came from the UK with the remainder working in Denmark (23%, *n* = 9), India (*n* = 1) and Malaysia (*n* = 1) surgeons.

**Fig. 1:**
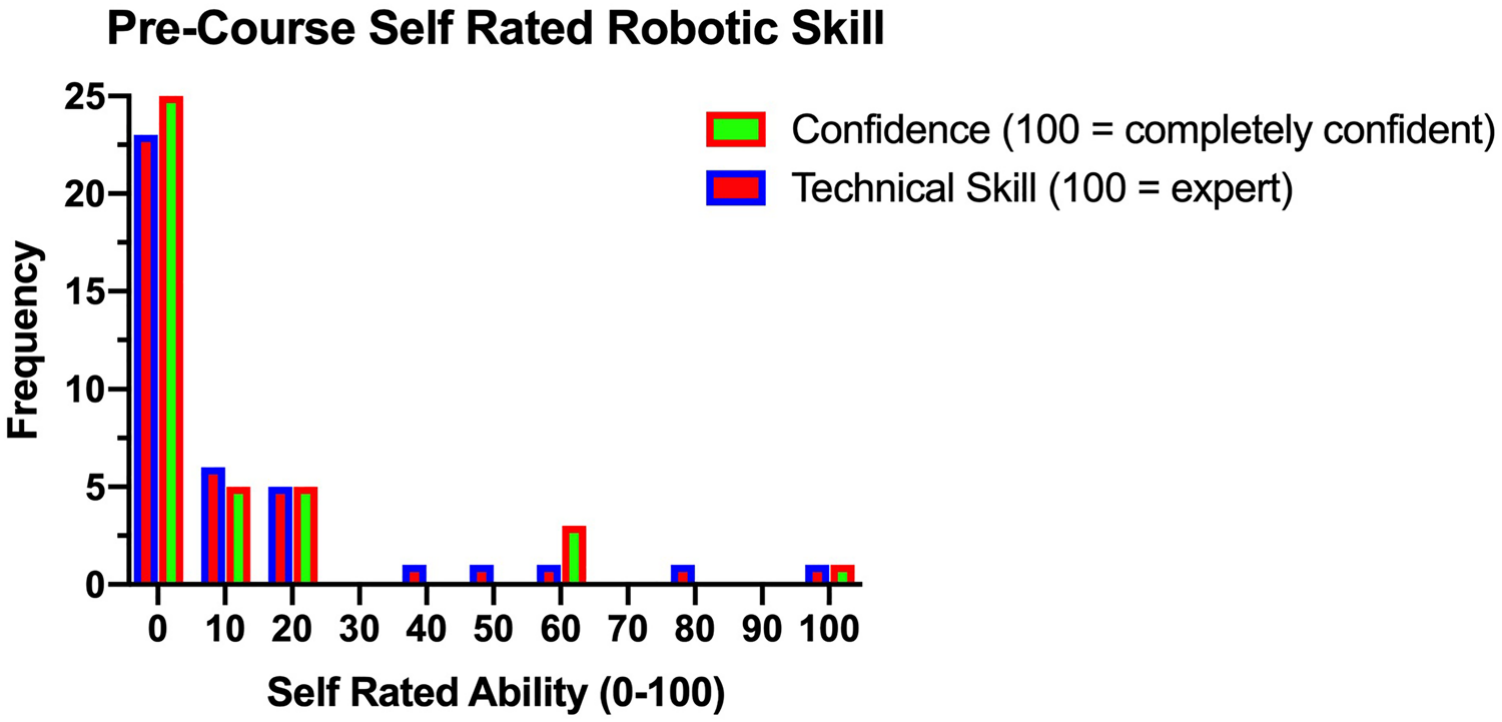
Participants’ pre-course self rated robotic skill

**Fig. 2:**
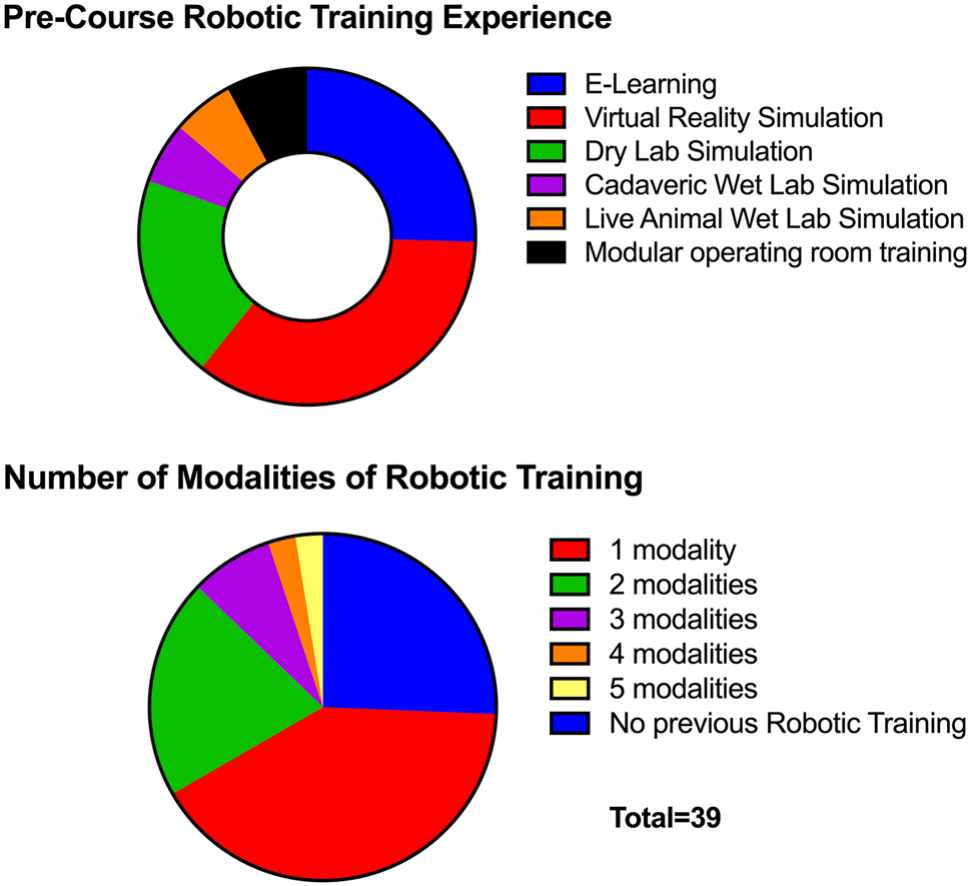
Pre-course participant robotic training experience

Self-rated robotic technical skill and confidence were both low before the course (mean 3.5 ± 6.8 and 3.5 ± 7.4, respectively) (Fig. 1). Similarly, most participants had no console experience prior to the course (median number of cases of console experience 0, range 0–100). 74% of participants (*n* = 29) had undertaken some form of robotic training before the porcine robotic course. 41% (*n* = 16) used just one form of training (Fig. 2).

**Fig. 3:**
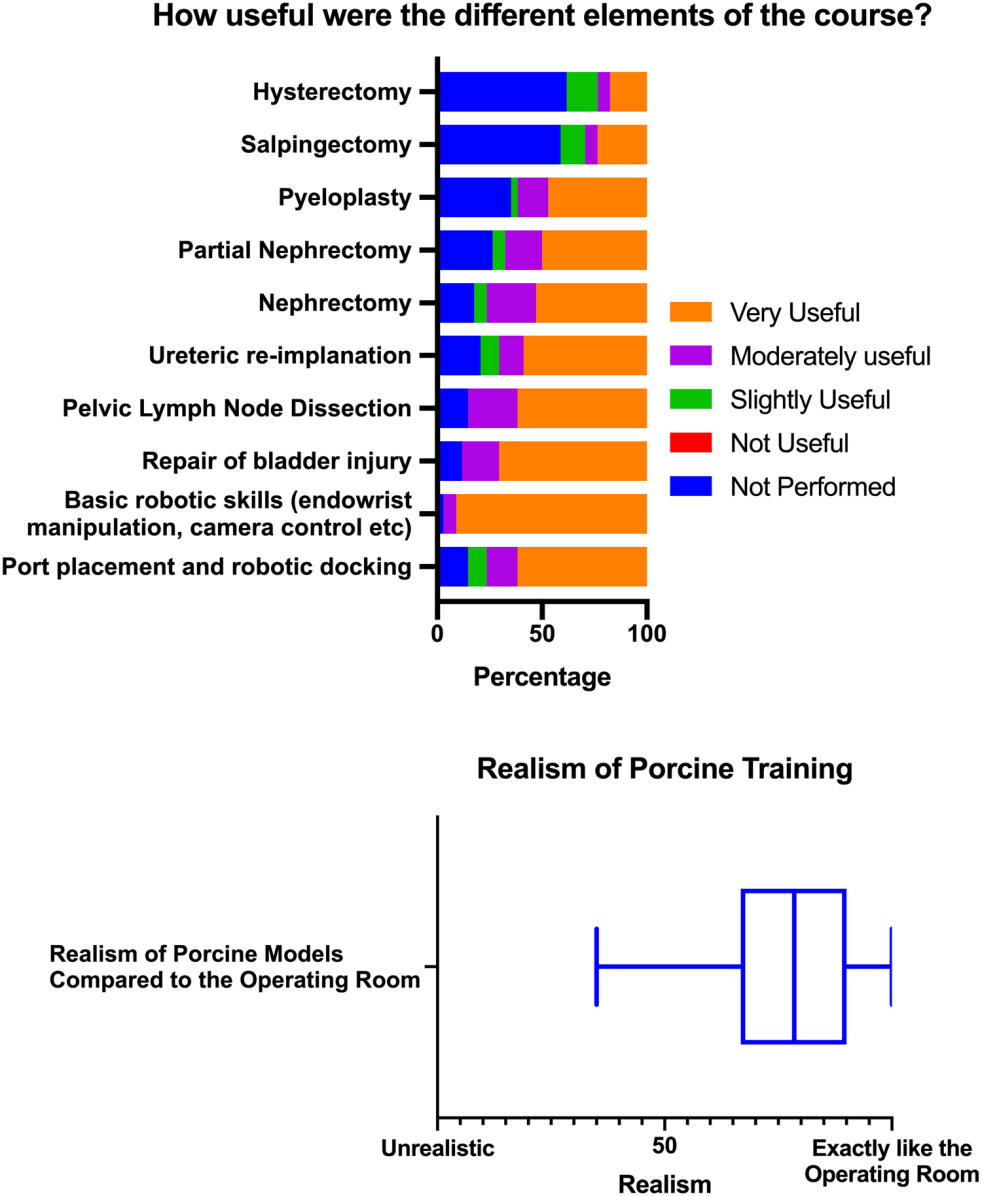
Perceived usefulness and overall realism of live porcine procedural simulation

Participants were questioned on the perceived usefulness, effectiveness and realism of the training course. Responses regarding both the individual course components and porcine training in general were overwhelmingly favourable (Fig. 3). The most useful were port placement and docking, basic robotic skills training and repair of a bladder injury.

**Fig. 4:**
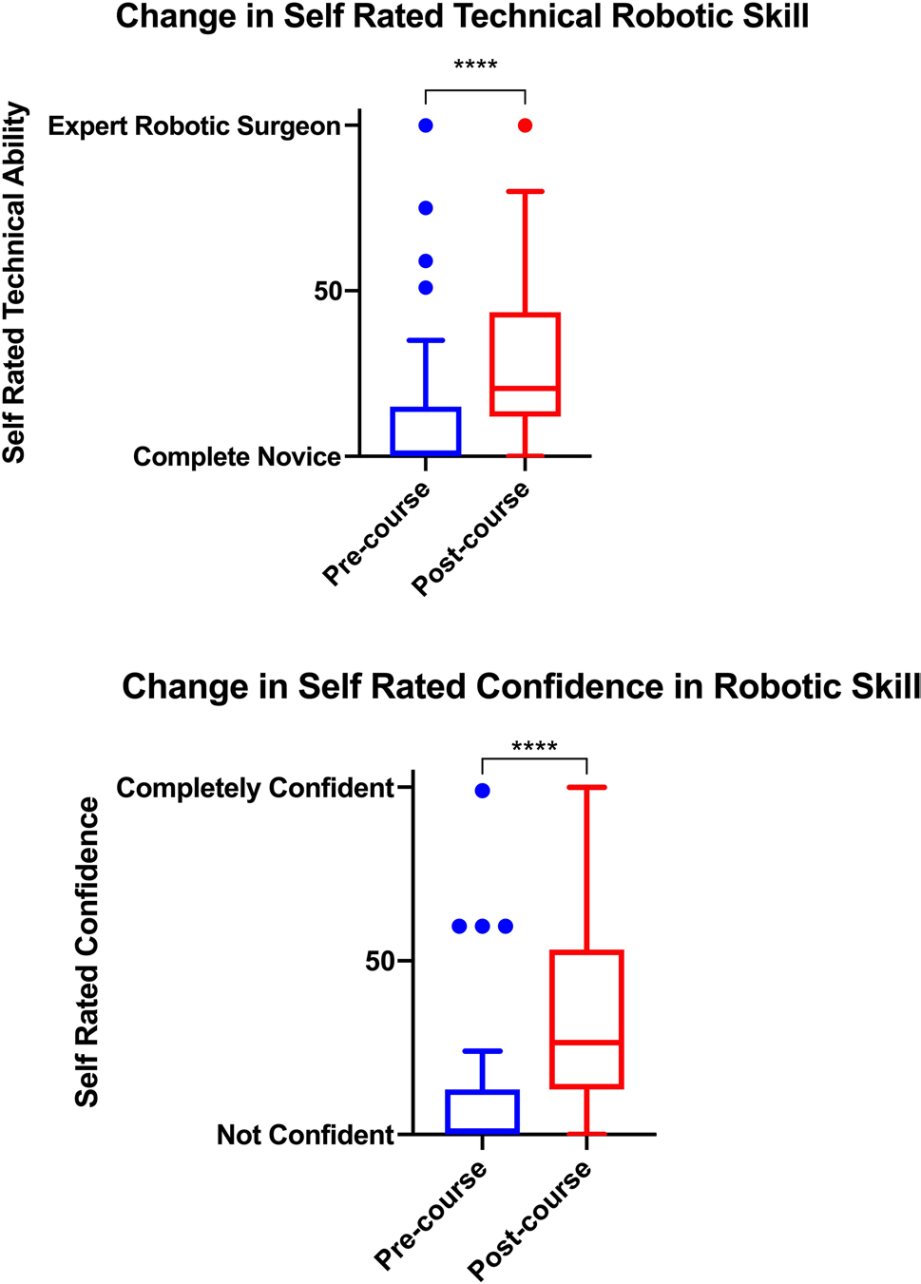
Change in self rated ability and confidence

Participants were asked to rate their own robotic surgical ability and confidence in performing robotic surgery following the training course. 72% of participants (*n* = 28) reported an increase in their perceived ability and 79% (*n* = 31) reported an increase in confidence in their robotic skill. Overall there were statistically significant increases in both self-rate technical ability and confidence following the course (Fig. 4). The relationship between self-assessed technical ability/confidence post training and pre-course technical ability/confidence and robotic surgical experience was modelled using multiple regression analysis. For self-assessed technical ability, there was no relationship to pre-course self-assessed ability (*p* = 0.06) but robotic experience did significantly contribute to post course self-assessed technical ability (*p* = 0.05). In contrast post-confidence was related to pre-course confidence (*p* = 0.005) but not robotic experience (*p* = 0.24).

The final questions in the survey regarded the participants further robotic training and practice after the course. The majority of participants took part in further training after completing the Aalborg Programme (*n* = 29, 74%) and 49% continued to either train or perform robotic surgery after the course.

## Discussion

A cross-sectional survey was used to analyse both the population of surgeons undertaking live animal robotic surgical training and the effectiveness of the training programme. The study has highlighted a number of important considerations for porcine robotic training. Despite the course providing relatively advanced robotic skills training, itself a subspecialist surgical field, the majority of participants were in the early stages of their residency training. This was further reflected in low pre-course skill and robotic surgical experience. Trainees had completed limited amounts of robotic training prior to the course. Over 25% of participants (*n* = 10) had no robotic simulation experience. 41% (*n* = 16) had used only one simulation training modality of which 6 participants had used only e-learning and undertaken no practical training. In total 16 participants (41%) had not previously undertaken any hands-on training in robotic surgery and had a median console experience of 0 robotic cases.

Despite low levels of prior experience, participants overwhelmingly scored the procedural training as very useful. Interestingly basic skills training was highest scored and was the most frequently performed activity, completed by 38/39 participants. Of the procedural training components, repair of bladder injury, pelvic lymph node dissection and ureteric reimplantation were considered to be the most useful. Nephrectomy, salpingectomy and hysterectomy were scored the lowest. The low scores of the latter two procedures were likely due to majority of participants being urologists and experience in these procedures offers little utility. The realism of porcine training was recognised by the majority of participants. This further reflected in the significant improvements seen in self rated technical ability and confidence following the course. Yet in spite of the above and whilst the majority of participants (71%, *n* = 24) under took further robotic simulation training, just under half of participants continued to train or practice in robotic surgery.

Live animal simulation has considerable limitations. The main barriers to undertaking such training are the costs and capital requirements necessary to support an animal laboratory. As discussed above full veterinary support is indispensable. From a practical point of view, an appropriate venue is also important. Whilst simulation laboratories are often relegated to a dark corner of the hospital, for animal training well ventilated and spacious premises are required [3]. As a result, it is important to establish the unique benefits of using live animals over other forms of training. The advantages of responsive tissues and the ability to manage complications are not relevant to all trainees. For initial basic training, the emphasis lies on gaining the correct psychomotor skills that can be effectively provided with benchtop models. As our results show, porcine training is attractive to surgeons of all experiences. However, to ensure the optimal use of porcine models, restricting trainees to those with more experience may need to be considered. Combining live porcine training with basic skill workshops using benchtop stimulators could also be considered to allow participants to gain a minimum level of technical ability before the course. It is not surprising that studies of live animal training frequently high satisfaction scores; the ability to practise on a live animal is clearly attractive [4, 5]. Shetty et al. postulated that differences were due to simpler dry and VR models being less stimulating. It is therefore important to note the lower satisfaction scores of the nephrectomy training in particular. This is likely to be due to anatomical variations in swine. The absence of retroperitoneal or perinephric fat in pigs means that nephrectomy is substantial easier to perform than in humans. A study comparing cadaveric to live animal urological training reported that human cadavers were preferred over live animals [6].

Limitations to this study should be considered. A relatively large cohort of participants were included in the survey in comparison with similar studies [7, 8]. Yet the low response rate means selection bias needs to be taken into account. Attempts were made to counter and reduce potential bias; the survey was designed to avoid leading questions and required a variety of response types to prevent acquiescent responses. The high rate of responses to blank box questions supports the active participation of respondents in this survey and the value of the results. Self-reported outcomes are also not equitable to objective assessments of training outcomes but do provide useful insights in the perceived usefulness and acceptability of training. Finally, as the survey included participants from a number of years, recall bias needs also to be considered.

## Conclusion

This cross-sectional survey of live porcine robotic training demonstrates it to be a widely valued, acceptable and feasible form of advanced robotic skills training. Despite the relative inexperience of participants prior to undertaking the course, significant improvements in self-assessed outcomes were reported. This was mirrored by high rates of further training and robotic surgical experience undertaken by the trainees. The results of this survey support the use of live porcine training for robotic surgery. Limitations principally due to the cost and infrastructure required to provide mean that careful consideration is required for its implementation in robotic training programmes. Training is best delivered to intermediate and advanced robotic training to ensure the unique benefits of live animal training are realised.

## Electronic supplementary material

Below is the link to the electronic supplementary material.

Supplementary Fig. 1: Aalborg participant survey questionnaire (PDF 205 kb)
