# Supplementary material for: An evaluation of live porcine simulation training for robotic surgery
Source: J Robot Surg. 2020 Jul 11;15(3):429–34. doi: 10.1007/s11701-020-01113-3 (PMC8134281; doi:10.1007/s11701-020-01113-3)
Supplement: Supplementary file 1 — Supplementary Fig. 1: Aalborg participant survey questionnaire (PDF 205 kb) [file 11701_2020_1113_MOESM1_ESM.pdf]

Live Porcine Simulation Training for Robotic Surgery:
The Aalborg Experience

1. Level of training at the time of completing the Aalborg course (most recent if multiple visits)

2. Your Speciality

3. In which country do you practice ?

4. Before completing the course, how would you have rated your robotic surgical skill?

Complete novice — Expert robotic surgeon

5. Before completing the course, how confident were you in performing robotic surgery?

Not at all confident — Completely confident

6. Before the course, approximately how many robotic procedures had you been involved in as console surgeon?

0 — 100+

7. Before the course, had you undertaken any other training in robotic surgery?

- [ ] e-Learning
- [ ] Cadaveric training
- [ ] Virtual Reality Simulation
- [ ] Live animal training
- [ ] Robotic training using plastic models
- [ ] Modular operating room training

8. Following the course, how would you rate your surgical ability?

Complete novice — Expert robotic surgeon

9. Following the training course, how confident did you feel in your robotic surgical skill

Not al all confident — Completely confident

10. Overall how would you rate the educational benefit of the course?

Not beneficial — Extremely beneficial

11. How effective do you think the live porcine robotic course was in training robotic skill?

Not at all effective — Extremely effective

12. How useful were the different modules of the course?

	Not Performed	Not useful	Slightly useful	Moderately useful	Very useful
Port placement and robotic docking	○	○	○	○	○
Basic robotic skills (endowrist manipulation, camera control etc)	○	○	○	○	○
Repair of bladder injury	○	○	○	○	○
Pelvis Lymph Node Dissection	○	○	○	○	○
Ureteric re-implanation	○	○	○	○	○
Nephrectomy	○	○	○	○	○
Partial Nephrectomy	○	○	○	○	○
Pyeloplasty	○	○	○	○	○
Salpingectomy	○	○	○	○	○
Hysterectomy	○	○	○	○	○

13. How realistic was training on the porcine models (appearance and tissue characteristics) in comparison to the operating room

Not at all realistic — Exactly like the OR

14. Training on porcine models taught useful skills that were transferable to the operating room

Completely disagree — Completely agree

15. Following this course, what further robotic training have you undertaken (please select all applicable)?

- [ ] I had no further robotic training
- [ ] Cadaveric Training
- [ ] Virtual Reality Simulation Training
- [ ] Live animal training
- [ ] Training with the robotic on plastic models
- [ ] Operating room training

16. How could the course be improved?

17. Please give any further comments on the training received

18. Do you currently perform robotic surgery?

- ○ I no longer perform robotic surgery
- ○ I am currently training in robotic surgery
- ○ I perform robotic surgery independently
